# Antidepressant Use in Medicaid-Insured Youth: Trends, Covariates, and Future Research Needs

**DOI:** 10.3389/fpsyt.2020.00113

**Published:** 2020-03-13

**Authors:** Julie M. Zito, Dinci Pennap, Daniel J. Safer

**Affiliations:** ^1^ Department of Psychiatry, University of Maryland, Baltimore, MD, United States; ^2^ Department of Pharmaceutical Health Services Research, School of Pharmacy, University of Maryland, Baltimore, MD, United States; ^3^ Center for Drug Evaluation and Research, United States Food and Drug Administration (FDA), Silver Spring, MD, United States; ^4^ Johns Hopkins Medical Institutions, Baltimore, MD, United States

**Keywords:** antidepressants, polypharmacy, children, adolescents, Medicaid, foster care, trends

## Abstract

**Background:**

Detailed research on long-term antidepressant (AD) trends within a single large US Medicaid population of youth has not heretofore been reported.

**Methods:**

Administrative claims data for eight annual timepoints across 28 years (1987–2014) were organized for youth (<20 years old) who were continuously enrolled during each study year in a mid-Atlantic state Medicaid program. Total annual AD prevalence and age-, gender-, race-, eligibility group-, and diagnosis-specific prevalence were formed from bivariate analyses; logistic regression assessed the change in use (2007–2014) adjusted for covariates. AD-polypharmacy data were assessed in 2014.

**Results:**

The major findings are: 1) AD use in state Medicaid enrollees grew 14-fold between 1987 and 2014. Data from 2014 revealed significantly increased odds of youth with SSRI/SNRI dispensings compared to 2007 (AOR=1.15 95% CI 1.11–1.19), representing 78% of total AD users. 2) Recent AD increases were greatest for 15–19-year olds. 3) AD use in girls passed up AD use in boys for the first time in 2014. 4) In 2014, ADs for foster care (12.7%) were 6 times greater than for their income-eligible Medicaid-counterparts. 5) In 2014, a quarter of AD-medicated youth were diagnosed with a behavior disorder. 6) More than 40 percent of AD medicated youth had >=1 other concomitant psychotropic classes for 60 or more days.

**Conclusions:**

Second-generation antidepressant use in Medicaid-insured youth has increased despite growing questions that pediatric AD benefits may not outweigh harms. These patterns support the call for publicly funded, independent investigator-conducted post-marketing outcomes research.

## Introduction

Since the introduction of second-generation antidepressants (selective serotonin and selective norepinephrine reuptake inhibitors [SSRI and SNRI]) in 1988, there has been a steady increase in their use to treat major depressive disorder and other mental health conditions in the pediatric population. Olfson et al. found parent-reported antidepressant (AD) prevalence doubled from 7.0% to 14.8% from 1996–1999 to 2010–2012 in a national survey of 6 to 17-year olds (1). The expanded use of antidepressants for adolescents was corroborated in another national survey with data from 2005 to 2014 showing a significant recent growth of one-third more medicated youth among 12 to 17-year olds (2). Over the years since their introduction to the market, SSRI/SNRIs have gradually constituted an increasing proportion of antidepressants prescribed for the treatment of pediatric depression (3).

Despite expanded use, controversy plagues the benefit-risk discourse on AD use for youth. Questions persist regarding AD efficacy (4), the 2004 FDA boxed warning and safety concerns for suicidality (5), new questions about long-term use with the risk of weight gain and metabolic disorders (6) and prominent concerns about difficulty to discontinue ADs in long-term users due to a withdrawal syndrome (7). Added to these concerns is the fact that pediatric AD use is largely off-label, that is without adequate evidence that benefits outweigh risks either because of age or the diagnosis and indication selected by the treating physician (8). Off-label AD use is particularly acute in the pediatric population. Lee et al. showed that in a national study of youths 6 to 18 years of age in outpatient care settings from 2000 to 2006, antidepressants for an FDA-approved indication applied in only 9.2% of visits, the remainder being for off-label uses (9).

This paper seeks to increase awareness of the expansion of AD treatment, particularly SSRI/SNRI subclass use in youth and its relevance to evidence-based treatment. Initially, we report pharmacoepidemiologic trends and patterns of AD use across 28 years in publicly-insured youth. AD use is assessed for key covariates among selected subpopulations. Clinician-reported diagnostic patterns and AD subclass patterns are presented. Second, we assess the odds of receiving an AD dispensing in 2014 compared with 2007 adjusting for key sociodemographic, administrative and clinical covariates. Third, we offer recommendations for future research to address the dearth of outcomes of long-term AD use in community-treated youth.

## Materials and Methods

### Data Source

Medicaid data on youth from a mid-Atlantic state were organized in eight annual data extracts across 28 years from calendar year 1987 through 2014. In that time, the eligible youth population (0–19 years old) increased from 138,018 in 1987 to 538,901 in 2014. (The expansion in the insured population is partly explained by the 1997 legislation on the Children’s Health Insurance Program.) Datasets were assembled from enrollment files and administrative claims data (dispensed medications and, in 2007 and 2014, physicians’ files). The study years were selected from the authors’ prior published studies of psychotropic medication use and an unpublished dissertation study (10): 1987, 1991, 1996, 2001, 2006, 2007, 2010, and 2014. Collectively, the data points pinpoint antidepressant patterns across the years in a single system for state-wide Medicaid youth enrollees who were continuously enrolled for 10 to 12 months per year and appraised annually in each study year.

Retrospective analysis of eight separate datasets was undertaken; several analyses with enriched variables, e.g., diagnosis and AD polypharmacy, were available in 2007 and 2014. To examine changes in utilization, we constructed time trends based on cross-sectional annual prevalence data for the selected study years. The study received expedited status by the university review board.

### Study Measures

Measurements and analyses fall into three broad categories and were operationalized as follows.

#### Prevalence and Patterns of Antidepressant Use

Total AD prevalence was defined as the frequency of continuously enrolled youth with one or more claims for an AD dispensing in each study year per 100 eligible enrollees. Antidepressants were divided into three subclasses: selected serotonin reuptake and serotonin/norepinephrine reuptake inhibitors (SSRI/SNRI); tricyclic antidepressants (TCAs) and other antidepressants. NDC codes from pharmacy claims data were translated into drug names by a data dictionary. A list of drugs within subgroups is provided in **Supplemental Table 1**. Other psychotropic classes included stimulants; antipsychotics; anxiolytic/hypnotics; lithium and other mood stabilizers; alpha-agonists; atomoxetine (**Supplemental Table 2**).

Additionally, AD polypharmacy was operationalized in terms of the number of other psychotropic classes taken concomitantly with the AD in 2007 and in 2014. Concomitant medication use was defined as 60 or more days of overlapping days’ supply of AD with 1, 2, or ≥3 psychotropic medication classes. Thirty-day overlaps were examined for comparison.

#### Sociodemographic Correlates

Age-specific AD prevalence rates were established for the 4 age strata defined by US census categories (0–4, 5–9, 10–14, and 15–19). Age was categorized on the last day of the study year. Gender-specific AD prevalence is described in terms of the AD M:F prevalence ratio. Race/ethnicity is recorded from self-reported information and categorized as White; African-American; other or missing. Medicaid eligibility groups were categorized as foster care; disabled (received supplemental security income) and income eligible, i.e. family income below the federal poverty level (TANF) or within 200% to 300% of the poverty level (CHIP).

#### Diagnostic Correlates

Additional variables were available for the 2007 and 2014 datasets allowing information on clinical-reported diagnoses to be assessed among AD users. International Classification of Disease (ICD-9) codes of psychiatric diagnoses (290.xx-319.xx) in the study year were organized into 4 categories in a hierarchical approach to capture psychiatric diagnoses of AD users: any depressive disorder was first, followed by behavioral disorders (ADHD and disruptive disorders) followed by other mental disorders and those with no psychiatric diagnosis was last. Diagnostic frequency in the study year was measured as a percent of AD-medicated youth. A list of codes for each category is included in **Supplemental Table 3**.

### Statistical Analysis

Annual cross-sectional analyses were used to create population-based percentage prevalence for AD total (1987–2014) for time trends across 28-years, by subclass, and age-, gender-, race- and eligibility-specific AD use were calculated by year for the most recent period (2007–2014). Prevalence ratios according to gender (M:F) and for white: African American youth were calculated for selected years. For 2014 compared to 2007, a multivariate logistic regression model was used to estimate the odds of AD use with adjustment for age group, gender, race/ethnicity, Medicaid-eligibility category, and clinician-reported diagnosis. Finally, polypharmacy among AD users was assessed in terms of the number (and percent) AD regimens with 1 or more classes of other psychotropic medications. SAS v. 9.4 was used to conduct the analyses.

## Results

### Total Prevalence

**Figure 1:**
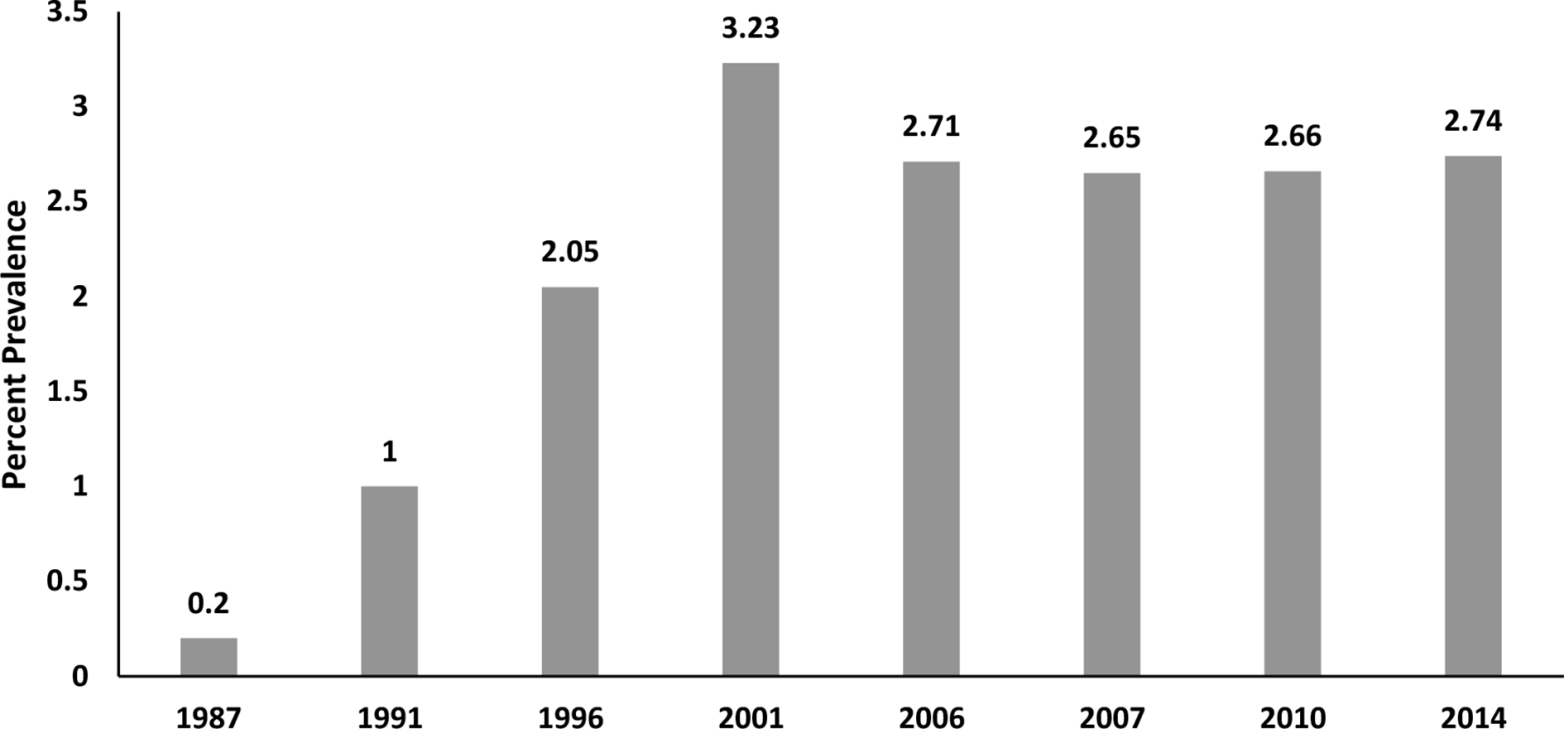
28-year Trend in Percent Prevalence of Antidepressants in Medicaid-insured Youth.

**Figure 1** shows the change in total AD use across 8 timepoints in 28 years. From a low of 0.2% in 1987, use rose in the 1990s to a high of 3.2% in 2001. However, rates began to decline following regulatory news in 2003 from the United Kingdom and then in 2004 from the FDA report on pediatric suicidality from clinical trial data showing an association with ADs (principally SSRI/SNRIs) (4). Antidepressant prevalence data from 2006 (2.71%) and 2010 (2.66%) are consistent with studies (1) showing fairly stable levels of AD use (compared with other psychotropic classes) after the FDA boxed warning in 2004. Across the 28 years, AD use grew from very low use to expand to many thousands more youth annually in this state Medicaid population—approximately 14-fold by 2014.

### Recent Antidepressant Subclass Patterns

Of the total 2014 AD use (2.74%), subclass analysis showed that the vast majority of AD use was for SSRI/SNRIs (2.14%). While SSRI/SNRI use was prominent, TCA use remained unchanged and other AD use dropped substantially. Other ADs included mirtazapine and trazadone. The subclasses sum to more than 100% because more than one subclass was dispensed to an individual (**Table 1**).

**Table 1:** Prevalence and Adjusted Odds Ratios for Antidepressant Use Comparing 2014 to 2007.

	2007		2014		
	N = 362,142		N = 538,901		AOR (95% CI)
	N	%	N	%	
Any AD	9,589	2.65	14,777	2.74	1.04 (1.01 – 1.07)
Any SSRI/SNRI	6,796	1.88	11,547	2.14	1.15 (1.11 – 1.19)
TCA	772	0.21	1,232	0.23	1.06 (0.97 – 1.16)
Other AD	3,334	0.92	4,206	0.78	0.86 (0.82 – 0.90)

Adjusted for age group, race/ethnicity, gender, Medicaid eligibility category, and psychiatric diagnosis.

### Odds of AD Use in 2014 Compared With 2007

**Table 1** also shows the adjusted odds ratio for any antidepressant of 1.04 (95% CI, 1.01–1.07) in 2014 which was slightly greater than in 2007. More robust is the increase in second generation ADs (SSRI/SNRI) (AOR = 1.15 95% CI = 1.11–1.19). Moreover, SSRI/SNRI users were the great majority of users, representing 78% of users.

### Age-, Gender-, Race- and Eligibility Group-Specific Patterns

**Table 2** features AD dispensing changes specifically within subpopulations across 8 years. Focusing on 2014 compared with 2007, age-specific AD patterns show a shift to older youth. Use in 0- to 9-year olds decreased since 2007 and nearly 8% of 15- to 19-year olds had an AD dispensing in 2014. Gender-specific ratios indicate that males were the predominant users in 2007 (M:F 1.13:1) but the ratio switched in 2014 to a slight predominance of females (M:F 0.9:1). Age and gender patterns before 1987 to 2006 are consistent with 2007. Race-specific data were measured as White/Black ratios showing more than twice as much use in White as in Black youth (2.76:1) in 2014. Compared with 2007, the ratio indicates an increase in 2014 for Black youth with AD dispensings. Substantial AD growth from 2007 to 2014 occurred in foster care youth exceeding even youth with disability (SSI covered youth). In 2014, foster care youth were 6 times more likely to receive an AD dispensing compared with their income eligible counterparts, i.e. those with poverty or near poverty family income.

**Table 2:** Demographic and Clinical Characteristics of Antidepressant Users Comparing 2014 to 2007 according to Row %.

	2007 (N = 362,142)		2014 (N = 538,901)	
	N	%	N	%
Age Group				
0 – 4	27	0.03	19	0.01
5 – 9	1,171	1.19	1,400	0.87
10 – 14	3,329	3.98	5,013	3.81
15 – 19	5,062	6.89	8,345	7.75
Gender				
Male	5,135	2.81	7,051	2.58
Female	4,454	2.48	7,726	2.91
Race/Ethnicity				
White	5,398	5.66	7,467	5.67
Black	3,573	1.85	4,874	2.05
Other/missing	618	0.83	2,436	1.44
Eligibility Category				
Foster Care	1,820	10.79	1,675	12.73
SSI (Disabled)	2,104	10.32	2,673	11.72
Income Eligible	5,665	1.74	10,429	2.07

Additionally, **Table 2** presents row percent data and shows AD use grew from 2007 to 2014 in foster care such that 12.7% of foster care youth had AD dispensings, an increase of 17.9%. By contrast, the analysis of column percent data (**Supplemental Table 4**) shows that foster care use of ADs dropped from 2007 to 2014. The drop, however, resulted from fewer foster care youth in total (denominator) rather than a drop in youth with dispensed ADs (numerator). Thus, analyses by row percent (**Table 2**) and column percent (**Supplemental Table, S.4**) were useful to clarify the patterns of AD users in foster care youth across the most recent 8-year period (available in terms of both subpopulations and time period). Furthermore, foster care prevalence exceeds the income-eligible prevalence by six-fold, a substantial difference.

### Clinician-reported Diagnostic Patterns

**Figure 2:**
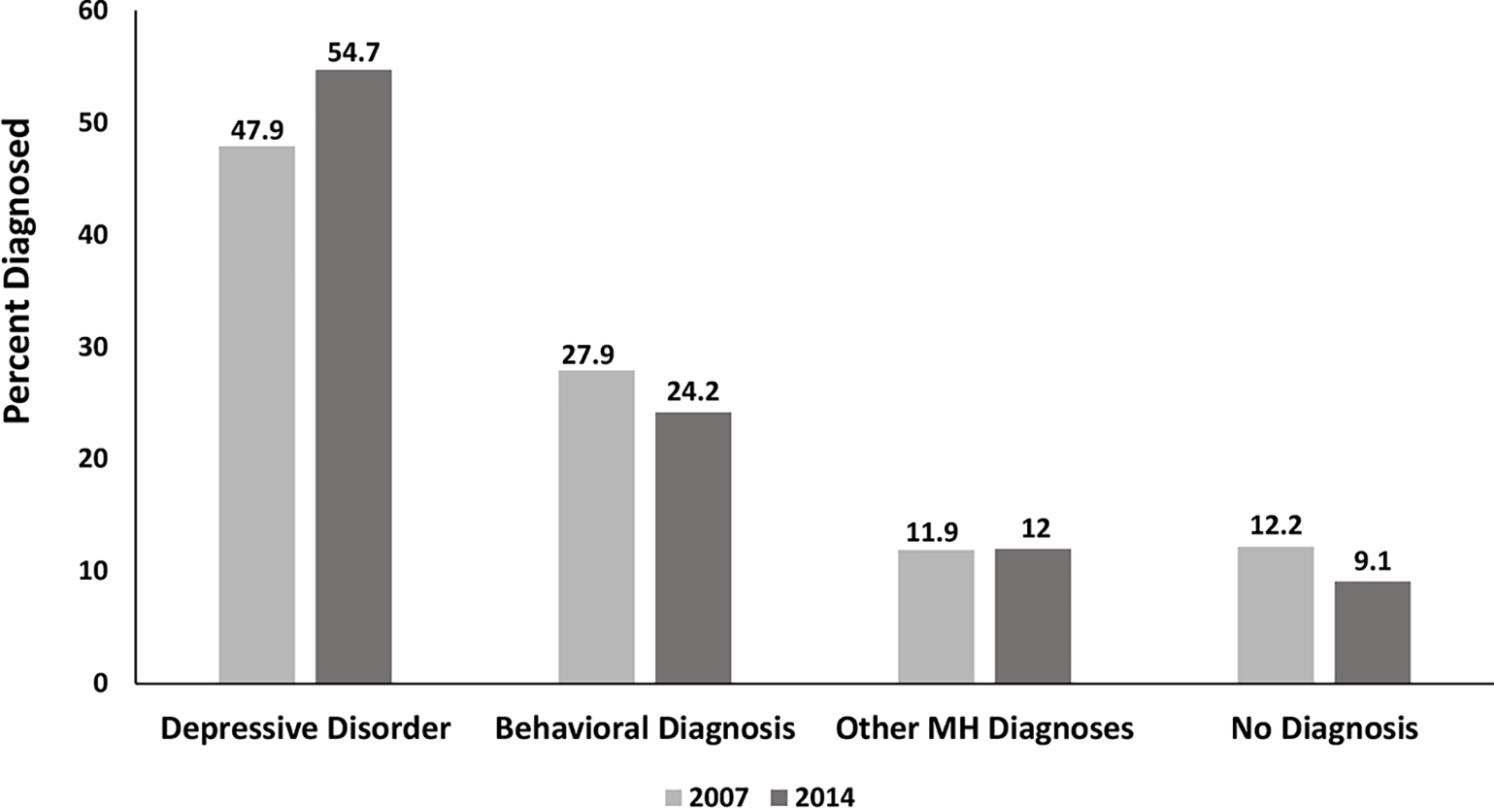
Clinician-reported Diagnosis among AD Users Comparing 2007 and 2014 Using Hierarchical Assignment. Behavioral diagnoses=ADHD and disruptive disorders; Other mental health diagnoses= all other diagnoses 295.xx – 319.xx.

**Figure 2** illustrates the ICD-9 clinician-reported diagnostic patterns according to the hierarchical assignment. Only half the diagnosed youth with AD dispensings had a diagnosis of a depressive disorder (DD). Behavior disorders were prominent for more than one quarter of youth and other mental health diagnoses accounted for the remaining 14%. Notably, 12.2% (2007) and 9.17% (2014) had no diagnosis associated with AD use.

### Antidepressant Concomitant (Polypharmacy) Patterns

**Table 3** reveals that monotherapy is the exception rather than the rule. Only 20% of AD use was for monotherapy in 2007 rising to 27% in 2014 using a ≥60 day overlap rule. Thus, concomitant psychotropic class use is widespread in this state Medicaid program. It is worthwhile to note greater proportions are defined as concomitant use when the window is only 30-days of overlap: the proportions are greater but switching cannot be ruled out.

**Table 3:** Distribution of Medication Use in 2007 and 2014 among Antidepressant Users According to Duration of Use and Regimen (Concomitant Use or Monotherapy).

	2007		2014	
	N	%	N	%
Total AD Users	9,589	100	14,777	100
Duration < 30 days	922	9.62	868	5.87
Duration ≥ 30 days	8,667	90.38	13,909	94.13
AD + ≥3 classes	665	6.95	1,075	7.27
AD + 2 classes	1,857	19.37	2,643	17.89
AD + 1 class	3,129	32.63	4,352	29.45
AD monotherapy	3,016	31.45	5,839	39.51
Duration < 60 days	3,401	35.47	4,573	30.94
Duration ≥ 60 days	6,188	64.53	10,204	69.06
AD + ≥3 classes	499	5.20	827	5.60
AD + 2 classes	1,400	14.60	2,104	14.24
AD + 1 classes	2,360	24.61	3,282	22.21
AD monotherapy	1,929	20.12	3,991	27.01

Regardless of duration of use (30 days or 60 days) or year (2007 or 2014), the top combinations were –

Combination of 4 or more:

Antidepressant + Alpha agonists + Antipsychotics + Stimulants

Antidepressant + Alpha agonists + Antipsychotics + Stimulants + Mood stabilizers

Antidepressant + Stimulants + Antipsychotics + Atomoxetine

Antidepressant + Alpha agonists + Antipsychotics + Mood stabilizers

Combination of 3:

Antidepressant + Antipsychotics + Stimulants

Antidepressant + Alpha agonists + Stimulants

Antidepressant + Stimulants + Mood stabilizers

Antidepressant + Alpha agonists + Antipsychotics

Combination of 2:

Antidepressant + Stimulants

Antidepressant + Antipsychotics

Antidepressant + Mood stabilizers

Antidepressant + Hypnotics

Antidepressant + Alpha agonists

### Specific SSRI/SNRI Medication Use

Up to 88% of SSRI users received a dispensing for sertraline, fluoxetine or escitalopram in 2007 which grew again in 2014 (**Table 4**). However, there were notable proportional decreases in venlafaxine, paroxetine, fluvoxamine, and duloxetine.

**Table 4:** Proportional Distribution of SSRI/SNRI medications for 2007 and 2014.

	2007 (N = 6,796 individuals)		2014 (N = 11,547 individuals)	
	N	%*	N	%*
Sertraline	2,082	30.63	4,562	39.51
Fluoxetine	2,251	33.11	4,138	35.84
Escitalopram	1,744	25.65	2,477	21.45
Citalopram	628	9.24	1,108	9.59
Venlafaxine	229	3.37	285	2.47
Paroxetine	289	4.25	240	2.08
Fluvoxamine	105	1.54	85	0.74
Duloxetine	159	2.34	126	1.09
Desvenlafaxine	0	—	22	0.19

*Percentages sum to more than 100 because more than one drug may be used in an individual.

## Discussion

It is worthwhile to note the antidepressant growth from 1987 to 2014 within a single system was 14-fold, a huge expanded use in Medicaid-insured children, largely for a relatively new molecular class of drugs (SSRI/SNRI). The gradual emergence of a distinct side effect profile in youth, different than the earlier widely used tricyclic antidepressants, is not surprising considering the rapid widespread adoption of second-generation antidepressants over 2+ decades. In 2014 compared to 2007, after accounting for age group, gender, race/ethnicity, eligibility group, and clinician-reported diagnosis, youth had modest but significantly increased odds of an AD dispensing. A more robust increase was observed for the SSRI/SNRI subclass.

In our data, recent increased use was almost entirely in 10- to 19-year olds with the majority in 15- to 19-year olds, perhaps suggesting a focus on the more traditional ages for depression. AD use in boys exceeded use in girls until 2014. Racial comparison of use was proportionally higher in white youth, although, notably, there was increased African-American use from 2007 to 2014. Clinician-diagnosed patterns suggest one-quarter of AD users were diagnosed with behavioral conditions, largely off-label conditions. Eligibility group data revealed that 12.7% of foster care youth received an AD dispensing in 2014, exceeding even those with federal disability status. The increased AD trend for foster care enrollees is consistent with an independently conducted analysis of this state’s pharmacoepidemiologic data from 2010 through 2013 (11).

### Foster Care Concerns

The prevalent use of ADs in the foster care population deserves attention. For the past decade, major federal agencies (12, 13) have conducted oversight medication studies of psychotropic use in foster care youth, calling attention to the extensive use of polypharmacy and the lack of monitoring that these high-risk medication regimens require. In the current study, there was a six-fold (5.98) greater AD use in foster care youth than in their family income-eligible counterparts. The deep disparity raises several questions: 1) Is it really illness severity that explains the disparity or unmet social needs? 2) Are state child welfare and judicial oversight programs responsible for requiring frequent physician visits which inadvertently increase multiple prescribers over time and could result in overtreatment with polypharmacy? Unfortunately, these questions cannot be addressed without new research to assess outcomes of treatment in community-treated youth. In particular, scientific approaches to safe reduction of dose and drug discontinuation in youngsters when complex multidrug regimens are ineffective would be most critical.

### Prevalence Estimates From Other Data Sources

Olfson et al. (1) traced the national growth in major psychotropic classes across 3 critical time periods from 1996 to 2012. Whereas a large increase in stimulant users and moderate growth in antipsychotic users were observed, antidepressant prevalence growth was more modest. AD prevalence was 2.5% in 2010–2012 among youth (6–17 years old) according to (parent-reported) data from the Medical Expenditure Panel Survey (1). Our Medicaid data for the closest matching age group (5–19 years old) was 2.64%. Narrowing the age range to adolescents, Mojtabai et al. (2) observed a prevalence of clinician-reported depression of 8.8% in 12- to 17-year olds with an increase in psychotropic medication for its treatment from 11.2% in 2005 to 13.6% in 2014 (2).

On the international level, Bachmann et al. compared pediatric AD use in a US-insured cohort with western European youth cohorts from four countries. In 2010, US percent prevalence was 1.3 to 3.2 times greater than that of Denmark, Germany, Netherlands, and United Kingdom in all age groups, (0–19 years), particularly among those less than 10 years of age (14).

### Polypharmacy

In the most recent psychotropic polypharmacy study in children with Medicaid insurance (fee for service type), multiclass polypharmacy applied to one-quarter of medicated youth less than 18 years of age (15). However, the operational definition of more than 1 psychotropic class from overlapping *dispensings* for 45 or more days was less restrictive than in previous studies and the current study (16). In a one-month extract of dispensings among Texas foster care youths, 41% of medicated youth experienced 3 or more concomitant classes (17). Antidepressants comprised 56% of these complex regimens. Comer and colleagues (18) operationalized psychotropic polypharmacy as 2 or more concurrent *prescription* orders in a national survey of physician office visits by 6- to 17-year olds. Their analysis revealed that among psychiatrically diagnosed youth, 2 or more classes grew from 22.2% (96–99) to 32.2%. (2004–2007). Our 2014 data for antidepressant polypharmacy for ≥60 days show substantial growth in polypharmacy compared with the Comer data. Comer measured any prescribed psychotropic combinations at one time whereas we assessed dispensed antidepressant combinations with 60 or more overlapping days. Methodologic variations limit precise comparisons, although the trends generally suggest increasingly complex regimens (2 out of 5 AD users) despite a dearth of evidence that polypharmacy benefits exceed risks.

In 2014, our analysis revealed that 40% of AD users had 1 or more other psychotropic classes in a 60-day window (**Table 3**) and many had a non-depression diagnosis. The combinations reflect a fundamental change in the theory of psychiatric treatment regarding medication use. Concomitant class use presents a non-specificity of psychopharmacologic class use regardless of diagnosis that is generally accepted as standard. In effect, theories of mental illness based on selection of specific classes of medication in relation to diagnosis are moot. Whereas the mechanism of action of a drug, e.g., fluoxetine was used in marketing campaigns tied to serotonin and norepinephrine brain receptor theory and promoted the concept that depression was a serotonin deficiency disorder (19), the theory does not explain the frequent use of ADs for behavioral disorders. Even less evidence exists for a 4-drug concomitant regimen of AD with stimulant, alpha-agonist, and antipsychotic, the most common 4-drug combination in the current data set. The drug-specific theory has been offered as an alternative to the diagnosis-specific theory (20). But, in our view, much stronger evidence is needed to accept multi-drug regimens in a drug-specific (i.e. symptom-specific) theory as the standard of care. The impact of polydrug interactions on the physical and mental health of youngsters is unknown and rarely the subject of clinical research.

### Emerging Risks of AD-Emergent Adverse Events

Like second generation antipsychotics, second generation ADs, specifically SSRI/SNRIs, have been the subject of serious adverse drug events, e.g., activation and recommendations for its clinical management (21). Earlier analysis of clinical trials has shown that SSRI adverse drug events (ADEs), e.g., activation and vomiting in children are two to three times more prevalent than in adolescents and least common in adults (22). As knowledge in adults has produced concerns about bone density changes and delayed sexual response, expanded, long-term use in adolescents demands a robust research agenda. Large simple trials would provide the population-based data that are needed while continuous monitoring of individual patients by the prescribing doctor who initiated the medication would aid in recognizing medication treatment-emergent symptoms. Risk assessment by Burcu et al. found the addition of ADs to an antipsychotic regimen increased the risk of type 2 diabetes two-fold among Medicaid enrollees from 5 large US states (23). The results were consistent when ADs were examined in a separate analysis (6). Risk increased with longer duration and higher dose in both studies (6, 23).

### Weak Effectiveness Findings

Meta analyses have cast doubt on the role of ADs alone for the treatment of pediatric depressive disorders. Cipriani et al. (24) conducted a network analysis of pediatric clinical trials to document benefits and risks from randomized double-blind clinical trials involving 5,260 youth and 14 ADs in terms of effectiveness and discontinuations due to adverse drug events (24). Only fluoxetine was significantly greater than placebo. Discontinuations due to ADEs were more likely for imipramine, venlafaxine, and duloxetine than placebo. The authors concluded that a clear AD advantage for children and adolescents was not shown. While we wait for clinical research to corroborate such big data analyses based on administrative claims data, we might consider revising medication authorization consent forms to include a statement that concomitant medication treatment has very weak research support.

In the current findings, it is encouraging to see reduced use of venlafaxine, paroxetine, fluvoxamine, and duloxetine as these shorter-acting products have been associated with more severe withdrawal (7), an important concern in balancing benefits and risks (24). Nevertheless, fluoxetine, the best evidenced SSRI represented only one-third of the leading SSRI usage.

### Future Research Directions

Our findings support the call for rigorous research on long-term benefit risk assessment in community populations including Medicaid-insured youth. These findings include: 1) The frequent use of ADs, particularly SSRI/SNRIs, has increased over the past 28 years even among less severely ill youths, i.e. those not meeting full criteria for major depressive disorder (25). 2) The relatively high off-label use has occurred despite growing concerns of weak AD effectiveness for major depressive disorder from meta-analyses. Equivocal effectiveness heightens concerns about unnecessary exposure to adverse drug events, e.g., weight gain and risk of type 2 diabetes mellitus (6, 23). In addition, the growth of polypharmacy (15) calls for scientific studies to safely reduce dosage and discontinue ADs safely. AD polypharmacy increases the risk of misdiagnosis of withdrawal symptoms upon discontinuation (26). 3) AD polypharmacy in the face of inadequate evidence of benefit increases the risk of unnecessary and costly adverse events (27).

More broadly, polypharmacy is a challenge in psychiatric practice because of the risk of not recognizing behavioral symptoms emerging from complex medication regimens and mistaking them for new underlying symptoms of illness—called behavioral toxicity (28). Behavioral toxicity is a relatively poorly recognized problem in psychiatry. Multiple prescribers increase the risk that patients will not be well known to each subsequent prescriber. Then, medications previously prescribed and responsible for new adverse symptoms will be viewed as new symptoms of illness to the prescriber who inherited a child on a complex concomitant regimen. Furthermore, a poorly designed health care delivery system makes continuity of care the exception rather than the rule for many Medicaid-insured youth (29). Moreover, failure to assure continuity of treatment puts patients at risk for unnecessary, costly services. Low continuity of care in the pediatric Medicaid population was shown to increase emergency department visits and psychiatric hospitalizations (29).

A critical priority for future research is post-marketing surveillance to ensure a long-term public health perspective on medication use. Such phase 4 research studies would go beyond the occasional FDA-mandated Risk Evaluation and Management Study (REMS) study conducted by manufacturers. REMS study results often have weak designs, delayed, or never completed and are rarely published in the clinical practice literature (30). If federal agencies (FDA, SAMHSA, and NIMH) prioritized funding for Phase 4 (post-marketing) studies, independent investigators could use advanced epidemiologic methods to follow cohorts over time so that ‘real world population’ use of medications would be comprehensively evaluated. Essentially, the expanded use of ADs, particularly SSRI/SNRIs, is a call for large national prospective cohort studies, possibly through electronic medical record studies at regionally diverse major academic centers. Protocols would be designed to assess functional improvement and safety outcomes in addition to assessing duration of treatment, monitoring for drug-induced risks, reasons for discontinuation, and following prescribed discontinuation regimens to minimize withdrawal syndrome.

### Limitations and Strengths

Administrative claims data though far from ideal provide inexpensive profiles of medication use in ‘usual care’ settings with ‘real world’ populations. This Medicaid study describes AD treatment in a single state Medicaid population over nearly 3 decades. Thus, the impact of geography and physician training are minimized as extraneous factors that might impact AD patterns over time. However, to our knowledge, the reach across 28 years in a single system is unique among pharmacoepidemiology studies. Furthermore, the medication patterns reflect dispensed medications so that unfilled prescription data do not inflate the prevalence. Nonetheless, the limitations of single state Medicaid prescription practice patterns include exclusion of US regional practices, limited ethnic subgroups, and coverage differences which may vary across state Medicaid programs. Selection of a Medicaid population may show greater prevalence of monotherapy and concomitant use if one uses federal oversight reports as a gauge of Medicaid drug utilization (12, 13). Privately insured patterns are much less available for comparison. Diagnostic data were not available before 2007. Furthermore, clinician-reported diagnoses are questionable as they do not rise to the level of research-assessed diagnoses, although learning how clinicians are linking drugs with diagnoses is useful. The study lacks information on the number and specialty of prescribers which did not permit continuity of care to be assessed.

## Conclusion

Second-generation antidepressant (serotonin and norepinephrine blockers) use in Medicaid-insured youth in this mid-Atlantic state has increased despite growing questions that pediatric AD benefits may not outweigh harms, in a vast increase in antidepressant use since 1987. These patterns support the call for publicly funded, independent investigator-conducted post-marketing outcomes research.

## Data Availability Statement

The datasets generated for this study are available on request to the corresponding author.

## Ethics Statement

The study was approved by the University of Maryland Institutional Review Board.

## Author Contributions

JZ and DS designed the study. DS critiqued the manuscript written by JZ. DP analyzed the data. All three revised text and contributed to the literature review.

## Conflict of Interest

The authors declare that the research was conducted in the absence of any commercial or financial relationships that could be construed as a potential conflict of interest.
