# Supplementary material for: Antidepressant Use in Medicaid-Insured Youth: Trends, Covariates, and Future Research Needs
Source: Front Psychiatry. 2020 Mar 13;11:113. doi: 10.3389/fpsyt.2020.00113 (PMC7082310; doi:10.3389/fpsyt.2020.00113)
Supplement: Supplementary file 1 [file DataSheet_1.docx]

**Supplemental Tables**

| **Table S1. Antidepressant Medications within Subgroups in 2014** | |
| --- | --- |
| **Subgroup** | **Medications** |
| SSRI/SNRI | citalopram, desvenlafaxine, escitalopram, duloxetine, fluoxetine, venlafaxine, fluvoxamine, paroxetine, sertraline |
| TCA | amitriptyline, amitriptyline/chlordiazepoxide, amoxapine, doxepin, imipramine, nortriptyline, protriptyline, clomipramine, desipramine |
| Other Antidepressants | Bupropion, trazodone, mirtazapine |

**Table S2. Other Psychotropic Classes**

| Medication Class | Generic Name |
| --- | --- |
| Alpha Agonist | clonidine, clonidine hcl, guanfacine hcl |
|  |  |
| Antidepressant | amitriptyline hcl |
|  | amitriptyline hcl/chlordiazepoxide |
|  | bupropion hbr, bupropion hcl |
|  | citalopram hydrobromide |
|  | clomipramine hcl, desipramine hcl |
|  | desvenlafaxine succinate |
|  | doxepin hcl, duloxetine hcl |
|  | escitalopram oxalate, fluoxetine hcl, fluvoxamine maleate |
|  | imipramine hcl, imipramine pamoate |
|  | mirtazapine, venlafaxine hcl, nortriptyline hcl |
|  | paroxetine hcl, protriptyline hcl |
|  | sertraline hcl, trazodone hcl |
| Antipsychotic | aripiprazole, clozapine |
|  | chlorpromazine hcl, fluphenazine hcl |
|  | haloperidol, haloperidol decanoate |
|  | fluphenazine decanoate |
|  | haloperidol lactate, thiothixene |
|  | loxapine succinate, olanzapine |
|  | olanzapine/fluoxetine hcl, paliperidone |
|  | perphenazine, pimozide, ziprasidone hcl |
|  | perphenazine/amitriptyline hcl |
|  | quetiapine fumarate, risperidone |
|  | ziprasidone mesylate |
|  | risperidone microspheres, thioridazine hcl |
| Atomoxetine | atomoxetine hcl |
| Hypnotic | buspirone hcl, chloral hydrate, clonazepam |
|  | diazepam, eszopiclone, lorazepam |
|  | midazolam hcl, zaleplon, ramelteon |
|  | zolpidem tartrate |
| Mood Stabilizer | carbamazepine, divalproex sodium |
|  | lamotrigine, oxcarbazepine |
|  | valproic acid |
|  | valproic acid (as sodium salt) (valproate sodium), lithium |
| Stimulant | dexmethylphenidate hcl |
|  | dextroamphetamine sulf-saccharate/amphetamine sulf-aspartate |
|  | dextroamphetamine sulfate |
|  | lisdexamfetamine dimesylate |
|  | methylphenidate, methylphenidate hcl |

| **Table S3. Psychiatric Diagnoses** | |
| --- | --- |
| Diagnostic Group | **ICD-9-CM codes** |
| Schizophrenia/other psychotic disorders | 295, 297-298 |
| Bipolar Disorder | 296.0, 296.1, 296.4 – 296.8, 301.13 |
| Intellectual disability | 317 – 319 |
| Autism Spectrum Disorder | 299, 299.8 |
| Disruptive disorders | 312.0-312.4, 312.81, 312.82, 312.89, 312.9, 313.81 |
| ADHD | 314 |
| Depression | 293.83, 296.2, 296.3, 296.9, 298.0, 300.4, 311 |
| Anxiety disorders | 293.84, 300.0, 300.2, 300.3, 308.3, 309.21, 309.81, 313.0, 313.2, 313.89 |
| Adjustment disorders | 308.0-308.2, 308.4, 308.9, 309.0-309.4, 309.82, 309.83, 309.89, 309.9 |
| Learning disorders | 307.0, 307.9, 315.0-315.2, 315.31, 315.32, 315.39, 315.9 |
| Other psychiatric disorders | 290-319 (includes only the codes not listed above) |

**Table S4.** **Demographic and Clinical Characteristics of AD Users Comparing 2014 to 2007** according to **column %**

|  | 2007 (N=9,589) | | 2014 (N=14,777) | |
| --- | --- | --- | --- | --- |
|  | N | % | N | % |
| Age Group |  |  |  |  |
| 0 – 4 | 27 | 0.28 | 19 | 0.13 |
| 5 – 9 | 1,171 | 12.21 | 1,400 | 9.47 |
| 10 – 14 | 3,329 | 34.72 | 5,013 | 33.92 |
| 15 – 19 | 5,062 | 52.79 | 8,345 | 56.47 |
| Gender |  |  |  |  |
| Male | 5,135 | 53.55 | 7,051 | 47.72 |
| Female | 4,454 | 46.45 | 7,726 | 53.28 |
| Race/Ethnicity |  |  |  |  |
| White | 5,398 | 56.29 | 7,467 | 50.53 |
| Black | 3,573 | 37.26 | 4,874 | 32.98 |
| Other/missing | 618 | 6.44 | 2,436 | 16.49 |
| Eligibility Category |  |  |  |  |
| Foster Care | 1,820 | 18.98 | 1,675 | 11.34 |
| SSI (Disabled) | 2,104 | 21.94 | 2,673 | 18.09 |
| Income Eligible | 5,665 | 59.08 | 10,429 | 70.58 |
| Diagnosis^ǂ^ |  |  |  |  |
| Depressive Disorder | 4,600 | 47.97 | 8,078 | 54.67 |
| Behavioral Diagnosis | 2,679 | 27.94 | 3,583 | 24.25 |
| Other MH* Diagnoses | 1,141 | 11.90 | 1.773 | 12.00 |
| No Diagnosis | 1,169 | 12.19 | 1,343 | 9.09 |

^ǂ^Hierarchical ranking starting with any depression (group 1); followed by any non-depression behavioral diagnosis (group 2); followed by other mental health diagnosis* (group 3). Behavioral disorders include ADHD and disruptive disorders. Other mental health (MH) diagnoses include all other codes in 295 – 319 than depressive and behavioral disorders.
